# A Rare Diagnosis in a Resource-Limited Setting: Alkaptonuria in a Young Dominican Child

**DOI:** 10.7759/cureus.92939

**Published:** 2025-09-22

**Authors:** Isi Y Ortiz Hernández, Cesarina García Grullon, Katherine Rijo Florimon, Lisa A Bueno Fernandez, Lisamelia Espaillat Solano

**Affiliations:** 1 Medicine, Pontificia Universidad Católica Madre y Maestra, Santiago de los Caballeros, DOM; 2 Pediatric Nephrology, Hospital Regional Infantil Dr. Arturo Grullón, Santiago de los Caballeros, DOM

**Keywords:** alkaptonuria, homogentisic acid, inborn errors of metabolism, ochronosis, pediatric case report

## Abstract

Alkaptonuria (AKU) is a rare metabolic genetic disorder transmitted in an autosomal recessive pattern caused by a deficiency in the activity of the enzyme homogentisate 1,2-dioxygenase (HGD), leading to progressive accumulation of oxidized homogentisic acid (HGA) as a dark pigment in connective tissues, cartilage, and organs in a process called ochronosis. Polymerization of HGA causes urine darkening after a few hours of exposure to the atmosphere, which is a hallmark of AKU. We present the case of a 3-year-old boy from a rural community in the Dominican Republic assessed at the pediatric hospital due to dark urine. The patient was directed to urology for suspicion of hematuria and later referred to the genetic pediatrics department in the absence of bleeding or urologic structural abnormalities. Afterward, a qualitative urine test and subsequent molecular testing via exome sequencing identified two heterozygous pathogenic variants in the *HGD *gene. Management consisted of a protein-restricted diet and vitamin C supplementation as an antioxidant. This case report emphasizes the importance of early detection and referral to a pediatric geneticist for prompt management, utilizing a multidisciplinary approach, to prevent complications and address the challenges associated with diagnosis and treatment in resource-limited settings and low-income families.

## Introduction

Alkaptonuria (AKU) is a rare metabolic genetic disorder and is one of the first diseases documented in medical history. Historical records date back to approximately 1500 BCE. The term “alkaptonuria” derives from the word “alkali,” referring to the ability of homogentisic acid (HGA) to absorb oxygen in alkaline media. The condition was first named by Boedeker in 1859 after observing reducing properties in the urine of a patient. However, it was not until 1891 that the identification of HGA as the compound responsible for the dark pigmentation in the urine in affected individuals occurred. The genetic defect was later mapped to chromosome 3q21-q23 in 1995 [1,2].

HGA is an intermediate metabolite in the amino acid catabolic pathway of tyrosine and phenylalanine. Biallelic mutations in the homogentisate 1,2-dioxygenase (*HGD*) gene, which encodes the HGD enzyme, impair the degradation of HGA and result in its progressive accumulation in various tissues. The accumulation leads to a process known as ochronosis, characterized by the deposition of a dark pigment in connective tissues, cartilage, and organs, ultimately causing multisystemic structural and functional damage [3-5]. Although AKU is rare, its global incidence is approximately 1 in 250,000 to 1,000,000 live births; certain regions demonstrate higher prevalence due to consanguinity or founder effects. These regions include Slovakia, India, and certain areas in the Caribbean, such as the Dominican Republic, where specific HGD gene mutations have been identified in local patients [4,6,7].

Clinically, the disease often goes unnoticed during childhood, with darkening of the urine being one of the earliest signs. However, musculoskeletal, cardiac, and dermatologic manifestations tend to develop later in life, once significant structural damage has occurred [4,8]. The diagnosis of AKU is based on elevated urinary HGA levels and the identification of pathogenic variants in the HGD gene. The availability of these tests has enabled the confirmation of cases that previously remained undiagnosed [3,6]. Despite advances in diagnostic testing, families from source-limited countries face financial difficulties in meeting dietary requirements and follow-up testing and appointments. This may result in inadequate management and the rapid progression of the disease.

This article presents a pediatric patient from the Dominican Republic with AKU, confirmed through both biochemical and genetic methods, along with a current review of the pathophysiology, diagnosis, and treatment of this rare disease, as well as the challenges associated with diagnosis and treatment in resource-limited settings and low-income families.

## Case presentation

We present the case of a 3-year-old boy from a rural community in the Dominican Republic who was referred to the pediatric department of Hospital Infantil Regional Universitario Dr. Arturo Grullón for evaluation of persistent urine darkening, first noted by his mother during the early months of life. The mother reports that the child’s urine appeared clear upon voiding but gradually turned dark brown after several hours, especially when left in the toilet bowl, and no associated urinary symptoms such as dysuria, hematuria, or polyuria.

**Figure 1:**
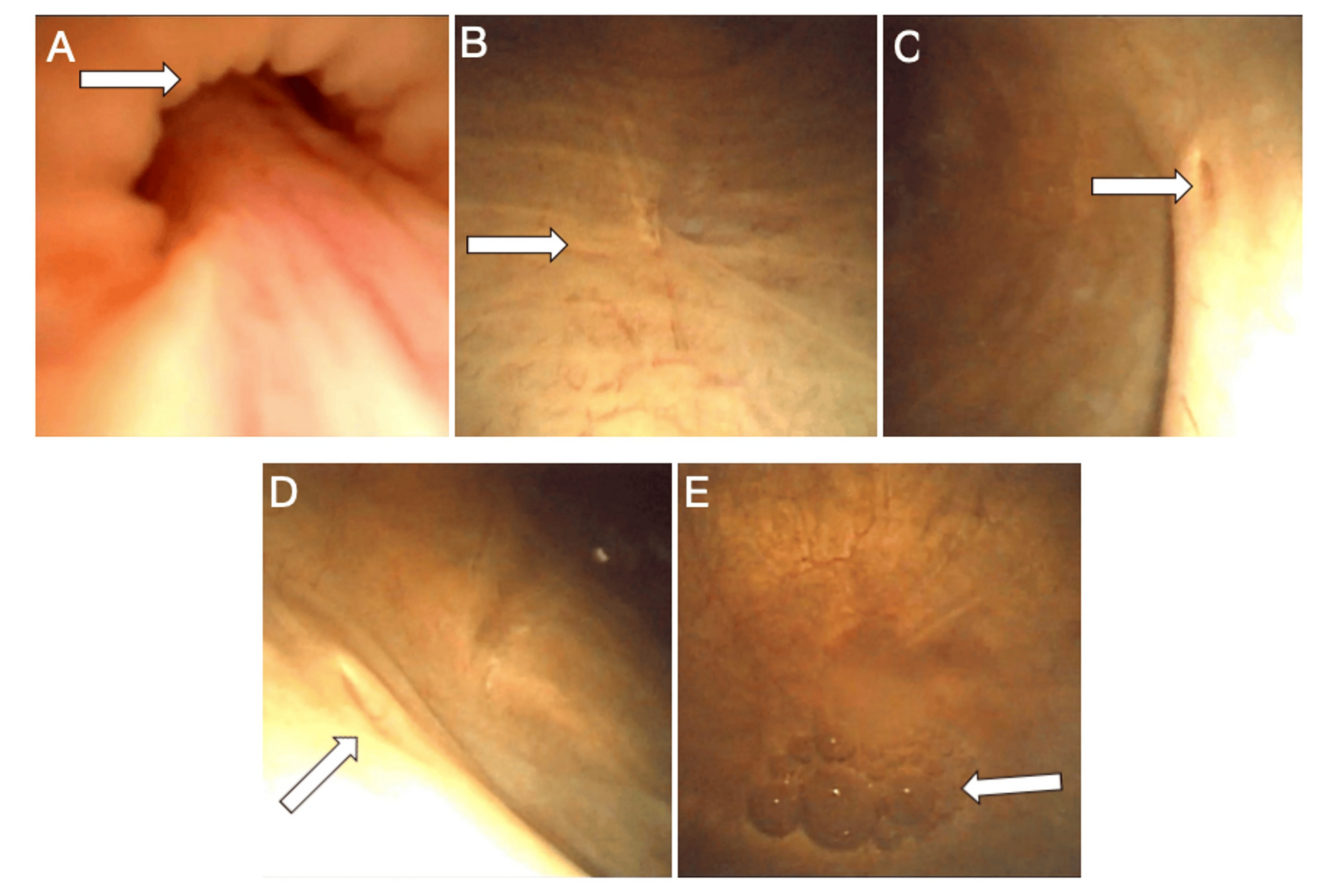
Normal cystoscopy findings A) Urethral sphincter; B) Trigone mucosa; C) Left ureteric orifice; D) Right ureteric orifice; E) Dome of the bladder

The first evaluation was conducted by a general pediatrician when the child was 1 year and 10 months old, who documented dark urine without accompanying urinary symptoms. Due to suspicion of hematuria, the child was referred to the urology department for further assessment, and cystoscopy was recommended as the most appropriate next diagnostic step. A cystoscopy was performed 6 months later and found to be negative for bleeding or structural abnormalities (Figure 1). Given the continued abnormal color of the urine in the absence of urological findings, a metabolic disorder was suspected, and AKU was considered in the differential diagnosis. Consequently, the pediatrician referred the patient to a pediatric geneticist for specialized evaluation.

The intake evaluation by the pediatric geneticist was performed four months later, consisting of a detailed patient history and physical examination, as described below. The child was born at term following an uncomplicated pregnancy and delivery. Growth and psychomotor development were appropriate for age. There was no history of joint pain, stiffness, limited mobility, or cutaneous abnormalities. The mother reports no family history of similar disorders, genetic or metabolic diseases, or parental consanguinity. The complete physical examination revealed no pathological findings in the musculoskeletal, dermatologic, cardiac, or respiratory systems. 

**Figure 2:**
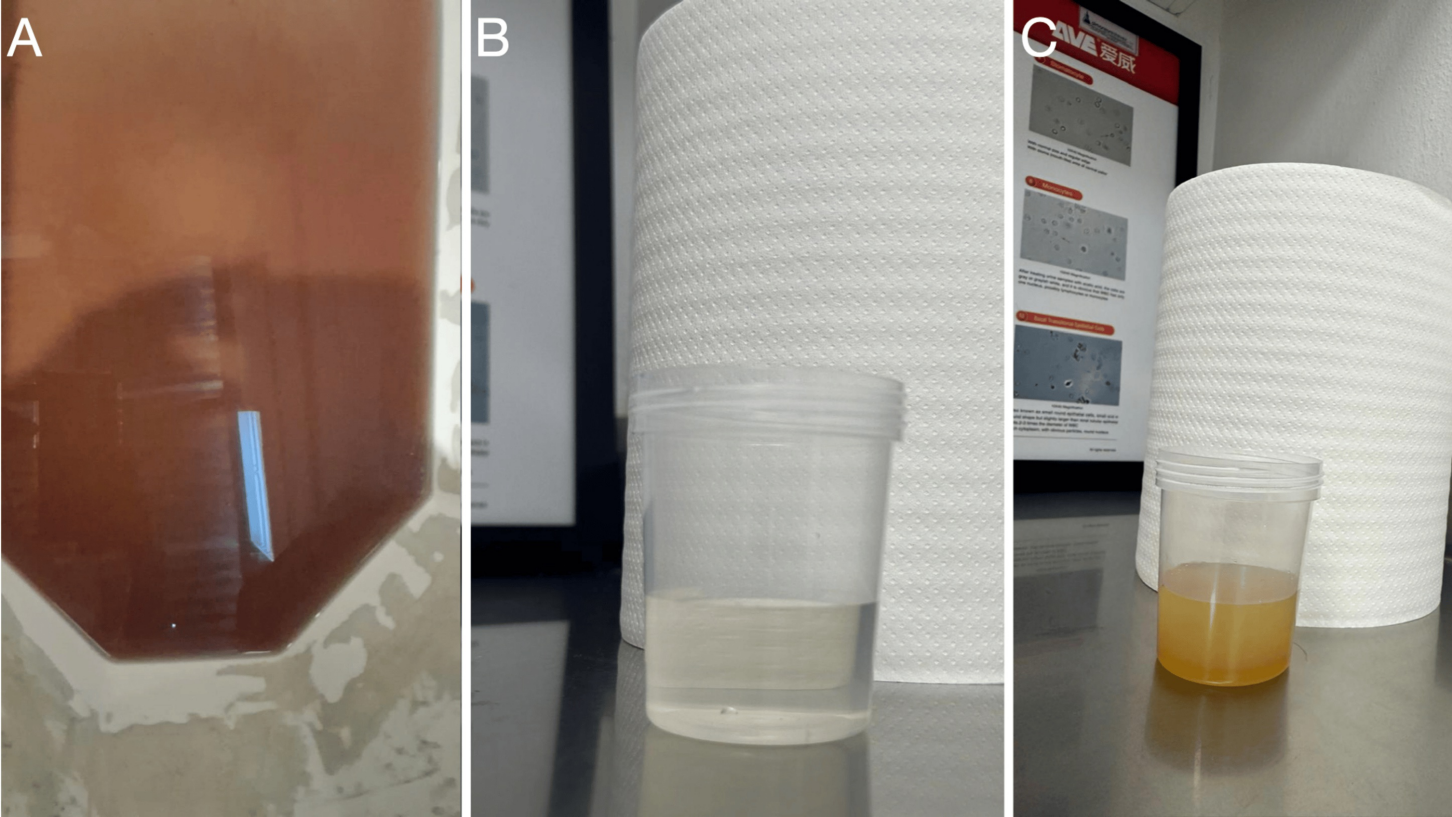
Urine samples (A) Dark brown urine sample, obtained via a urine collector during the initial evaluation, examined three hours post-collection. (B) Clear yellow urine sample, freshly voided during follow-up appointment after initiation of treatment. (C) Same specimen as (B) immediately after adding one drop of ferric chloride.

As part of the initial workup, a fresh urine sample was collected, which was initially clear but turned dark brown after 3 to 4 hours at room temperature, without foul odor or significant turbidity (Figure 2A). Based on these findings, molecular testing via exome sequencing was performed. The results in February 2025 identified two heterozygous pathogenic variants in the *HGD *gene: c.674G>A (p.Arg225His) and c.808G>A (p.Gly270Arg), both previously reported in patients with AKU. Although parental segregation analysis is still pending to confirm compound heterozygosity (trans configuration), the clinical presentation and biochemical findings support the diagnosis of autosomal recessive AKU.

The patient was referred for multidisciplinary follow-up with pediatric genetics and metabolic disease specialists. Supportive treatment was initiated, consisting of a protein-restricted diet to reduce tyrosine and phenylalanine intake, under strict nutritional supervision. Vitamin C supplementation was prescribed as an antioxidant. During the follow-up appointment and after proper treatment, a urine sample was collected (Figure 2B), and one drop of ferric chloride was added to it as an alkaline medium to potentiate the oxidation reaction. The test resulted in HGA generating pigmented products, which immediately turned the color of the specimen from clear to dark yellow (Figure 2C) instead of dark brown, demonstrating less HGA polymerization in urine. 

Accordingly, a panel of laboratory studies was requested, including a complete urinalysis, complete blood count (CBC), and comprehensive metabolic panel (CMP). All results were within normal limits, with no evidence of urinary tract infection, hematuria, electrolyte imbalance, or hepatic or renal dysfunction (Tables 1-2).

**Table 1:** Relevant laboratory results WBC: white blood cell count; RBC: red blood cell count; MCV: mean corpuscular volume; MCH: mean corpuscular hemoglobin; MCHC: mean corpuscular hemoglobin concentration; RDW-CV: red cell distribution width – coefficient of variation; MPV: mean platelet volume

Test name	Result	Reference Range
WBC	10.74	5.50 - 15.50 K/uL
RBC	4.48	3.90 - 5.30 M/uL
Hemoglobin	12.10	11.50 - 15.50 g/dL
MCV	83.48	75.00 - 87.00 fL
MCH	27.01	24.60 - 30.90 pg
MCHC	32.71	32.00 - 36.00 g/dL
Platelets	335.00	197.00 - 382.00 K/uL
RDW-CV	14.40	11.50 - 15.00 %
MPV	10.00	0.90 - 99.00 fL
Lymphocytes #	6.89	1.93 - 10.08 K/uL
Lymphocytes (%)	64.20	35.00 - 65.00 %
Neutrophils #	2.99	1.27 - 8.68 K/uL
Neutrophils (%)	27.90	23.00 - 45.00 %
Monocytes #	0.63	0.20 - 1.00 K/uL
Monocytes (%)	5.90	4.00-10.00 %
Basophils #	0.02	0.00 - 0.10 K/uL
Basophils (%)	0.20	0.00 - 1.00 %
Eosinophils #	0.19	0.00- 0.60 K/uL
Eosinophils (%)	1.80	0.00 - 3.00 %
Creatinine	0.25	0.50 - 1.50 mg/dL
Blood urea nitrogen	4.11	5.00 - 17.00 mg/dL
Cholesterol	131	0.00 - 200.00 mg/dL
Triglycerides	101.20	27.00 - 125.00 mg/dL
Glucose	87.45	70.00 - 110.00
Glycosylated hemoglobin	5.4	4.00 - 6.00 %
Uric acid	4.51	1.80 - 5.00 mg/dL
Calcium	10.14	9.00 - 11.00 mg/dL
Magnesium	2.01	1.50 - 2.50 mg/dL
Inorganic phosphorus	5.71	3.00 - 6.00 mg/dL

**Table 2:** Other relevant laboratory results

Parameter	Result	Reference Range	Interpretation
Urine color (fresh)	Light yellow	Light yellow	Normal
Urinary sediment	No pathological findings	No abnormal cells / elements	Normal
Cystoscopy	No evidence of hematuria / Normal bladder mucosa	—	Normal
Ferric chloride urine test	Positive (dark green)	Negative	Abnormal / Suggestive of homogentisic aciduria
Blood urea nitrogen	4.11 mg/dL	5–17 mg/dL	Slightly low (no clinical significance)

The patient is currently asymptomatic, with no joint manifestations, hyperpigmentation, or functional limitations. The mother reports feeling reassured and supported since the child began receiving specialized care, although she acknowledges ongoing financial difficulties in meeting some dietary requirements and additional testing. Despite these challenges, the family continues to comply with medical recommendations to the best of their ability, demonstrating resilience in the face of the diagnosis. 

## Discussion

AKU is a rare metabolic genetic disorder caused by mutations in the *HGD *gene that encodes the HGD enzyme. This enzymatic deficiency leads to systemic accumulation of HGA, which progressively deposits in connective tissues, cartilage, and organs, resulting in ochronosis and multisystem complications over time [3-5]. Diagnosis is often suspected in the presence of darkening urine upon exposure to air, a hallmark sign that may be evident from early infancy. However, this finding is frequently overlooked during the early stages, delaying clinical diagnosis until more overt symptoms, such as tissue pigmentation (ochronosis) or joint manifestations, emerge in adulthood [4,8,9]. The key biochemical marker is elevated urinary HGA, detectable through chromatography or mass spectrometry. This marker is highly sensitive and specific, allowing confirmation of the clinical suspicion [4,10]. In some cases, urine darkening intensifies upon exposure to alkalis, a valuable diagnostic clue in resource-limited settings [8,11].

Genetic testing through exome sequencing is also used to confirm the diagnosis of atypical AKU cases by identifying pathogenic biallelic mutations of the *HGD *gene. This analysis is also valuable in populations with high consanguinity or a positive family history, which facilitates early detection, intervention, and genetic counseling. Although reports of AKU in the Dominican Republic are scarce, a significant finding in 2009 identified a founder mutation, c.433G>A (p.Gly145Asp), in several unrelated patients. This variant has not been observed in other populations, which suggests a shared ancestral origin. These findings underscore the importance of targeted genetic testing in suspected cases, even in the absence of a family history, and highlight the need to expand access to molecular diagnostics in the country [7,8,10].

Although the natural history of the disease has been well described, therapeutic management has undergone considerable evolution in recent decades. Initially focused on symptomatic treatment, current strategies aim to alter HGA metabolism and slow disease progression [3,6,12]. Vitamin C has been widely used for its theoretical antioxidant effect, which may delay the conversion of HGA into ochronotic pigment. However, controlled studies have shown minimal or no impact on disease progression [4,6,9]. Nevertheless, due to its safety profile and low cost, it remains an attractive option in settings where other therapies are unavailable [6]. Dietary restriction of HGA precursor amino acids, particularly tyrosine and phenylalanine, has also been attempted to reduce endogenous HGA production. While this approach may slightly lower metabolic burden, its clinical impact has been modest. Moreover, in pediatric patients, it may pose nutritional risks if not adequately supervised [3,6,9].

The most significant therapeutic advance has been the introduction of nitisinone, an inhibitor of the enzyme 4-hydroxyphenylpyruvate dioxygenase, which acts upstream in the metabolic pathway to block HGA formation. Its use has significantly reduced urinary and plasma HGA levels and has been associated with clinical improvements in adults, particularly in joint pain, physical function, and progression of ochronosis. However, nitisinone utilization in pediatrics presents significant challenges, as pathway inhibition leads to elevated plasma tyrosine levels, potentially resulting in complications such as keratopathy and neurologic effects if not carefully monitored [3,6,9]. To date, the use of nitisinone in children remains under investigation, and expert recommendations vary. Some experts advocate for early introduction to prevent pigment deposition.

In contrast, others reserve it for patients with established clinical manifestations, given the lack of fully defined safety data in early life. Therefore, strict monitoring of tyrosine levels and periodic ophthalmologic and neurologic assessments are essential in pediatric patients receiving this treatment. Additionally, the use of nitisinone was recently approved by the FDA for AKU. Although the cost is high, some organizations provide it free of charge in certain countries. Nevertheless, because the drug is considered controlled, it requires close laboratory monitoring and specialized follow-up, which can be difficult in areas with limited resources [3,6].

## Conclusions

This case report underscores the importance of early clinical suspicion, prompt intervention, biochemical confirmation, and molecular diagnosis in identifying rare metabolic disorders, such as AKU. Even though genetic testing is beneficial in populations with high consanguinity and a positive report of the disease in the family, it is also crucial in highly prevalent regions where founder mutations have been identified as an ancestral origin, thus emphasizing the relevance of genetic testing in suspected cases, and the need to improve the accessibility of genetic testing in the country.

Furthermore, in pediatric populations, early recognition enables timely intervention and long-term monitoring by a multidisciplinary team, which may positively impact disease progression and patient quality of life. Additionally, the case highlights the importance of prompt referral to the genetics department in the presence of unusual or nonspecific clinical findings, such as persistent urine that darkens over time, to prevent diagnostic delays, optimize the initiation of individualized therapies, and avoid long-term complications. Finally, it is essential to recognize vital determinants, such as financial constraints in meeting dietary requirements, follow-up testing, and appointments, that will impact the proper management and progression of the disease. 
